# Premature ovarian insufficiency: a review on the role of oxidative stress and the application of antioxidants

**DOI:** 10.3389/fendo.2023.1172481

**Published:** 2023-08-01

**Authors:** Yu-Qian Shi, Xi-Ting Zhu, Su-Na Zhang, Yi-Fu Ma, Yan-Hua Han, Yue Jiang, Yue-Hui Zhang

**Affiliations:** ^1^ Department of First Clinical Medical College, Heilongjiang University of Chinese Medicine, Harbin, China; ^2^ Department of Obstetrics and Gynecology, Key Laboratory and Unit of Infertility in Chinese Medicine, First Affiliated Hospital, Heilongjiang University of Chinese Medicine, Harbin, China

**Keywords:** oxidative stress, aging and oocyte, premature ovarian insufficiency, antioxidant, mitochondrial dysfunction

## Abstract

Normal levels of reactive oxygen species (ROS) play an important role in regulating follicular growth, angiogenesis and sex hormone synthesis in ovarian tissue. When the balance between ROS and antioxidants is disrupted, however, it can cause serious consequences of oxidative stress (OS), and the quantity and quality of oocytes will decline. Therefore, this review discusses the interrelationship between OS and premature ovarian insufficiency (POI), the potential mechanisms and the methods by which antioxidants can improve POI through controlling the level of OS. We found that OS can mediate changes in genetic materials, signal pathways, transcription factors and ovarian microenvironment, resulting in abnormal apoptosis of ovarian granulosa cells (GCs) and abnormal meiosis as well as decreased mitochondrial Deoxyribonucleic Acid(mtDNA) and other changes, thus accelerating the process of ovarian aging. However, antioxidants, mesenchymal stem cells (MSCs), biological enzymes and other antioxidants can delay the disease process of POI by reducing the ROS level *in vivo*.

## 1 Introduction

POI is an important medical issue concerning women of reproductive age. It is a clinical syndrome defined by loss of ovarian activity before the age of 40 years. Over the years, several terms have been used to describe this condition, such as ‘primary ovarian insufficiency’, ‘premature menopause’ and ‘premature ovarian failure’. Although the latest guideline from the European Society of Human Reproduction and Embryology (ESHRE) adopted ‘premature ovarian insufficiency’ as the standard nomenclature (1). The diagnostic criteria are not fully standardized, yet the differences varied little. The latest diagnostic criteria proposed in the latest guidelines are primary or secondary amenorrhea or spaniomenorrhea of>4 months with onset before 40 year of age, and elevated follicle-stimulating hormone (FSH)>25IU/L on 2 assays at>4 weeks’ interval. Estradiol level is low, and anti-Müllerian hormone (AMH) levels have usually collapsed (2). The prevalence of POI varies among ethnic groups across the world, and studies show a prevalence that ranges from 1% (3) up to 5.5% (4). If not treated quickly, women living with POI face the short- and long-term consequences of prolonged hypoestrogenism and can develop symptoms such as lack of libido, vaginal dryness, mood disorders, rise in cardiovascular risk and cognitive impairment (5). Decreased mineral density promotes osteopenia and osteoporosis, as well as bone fractures. The health provider should be aware of POI signs and symptoms in order to promote prompt diagnosis (6). The most accepted ovarian aging theory emphasizes that the ability of ovarian GCs to counteract the ROS pathogenic effect is decreased (7), which ultimately leads to the reduction of the quantity and quality of follicles (8, 9). Reactive oxygen species (ROS) are two electron reduction products of oxygen, including superoxide anion, hydrogen peroxide, hydroxyl radical, lipid peroxides, protein peroxides and peroxides formed in nucleic acids (10). They are maintained in a dynamic balance by a series of reduction-oxidation (redox) reactions in biological systems and act as signaling molecules to drive cellular regulatory pathways (11, 12). The production of ROS can be induced by various factors such as heavy metals, tobacco, smoke, drugs, xenobiotics, pollutants and radiation (13). Excessive oxidative stress derived from ROS accumulation deregulates the antioxidative defense system, which is closely associated with various diseases (14, 15). ROS are produced in almost every subcellular organelle in the cell, including the plasma membrane, cytoplasm, mitochondria, core, endoplasmic reticulum, Golgi, and others (16). The main sources of cellular ROS are inflammation and mitochondrial dysfunction, as ROS are produced by immune cells in response to infection, injury and aerobic metabolism (17). In ovarian tissue, normal levels of ROS play an important regulatory role in follicular growth, intrathecal angiogenesis and sex hormone synthesis. Nevertheless, when oocytes are exposed to high concentrations of oxygen molecules or chemical derivatives of oxygen, they are susceptible to cellular damage, which may be manifested by impaired follicular oocyte development, POI and decreased reproductive function (18). The development of POI is associated with excessive accumulation of ROS in the ovary, which accelerates the aging of ovarian cells and reduces ovarian function (19). Studies have shown that perfluorononanoic acid (PFNA) can induce OS in oocytes, destroy spindle assembly, mitochondrial function and DNA structure, and induce apoptosis and hinder oocyte maturation *in vitro (* 20). In addition, high levels of OS-mediated ROS were also found to block GCs development and induce apoptosis in the hydrogen peroxide(H_2_O_2_)_–_treated POI mouse model, ultimately leading to follicular atresia (21, 22). As a product of OS, the pathological effect of ROS has been confirmed in many female reproductive diseases such as endometriosis, polycystic ovary syndrome, spontaneous abortion, hydatidiform mole and pre eclampsia (23). Therefore, it is of great significance to study the role of ROS, a product of OS, in the pathogenesis of POI for exploring new OS-targeting drugs.

## 2 The role of OS in POI

### 2.1 OS-mediated alterations of genetic material in POI

Recently, the second-generation sequencing has been used in large POI families to determine that the pathogenic factors mainly focus on DNA damage repair, homologous recombination, and meiosis (24). In this part, therefore, we discuss the role of OS-mediated DNA damage and alterations in genetic material including apoptosis, mtDNA dysfunction, meiotic abnormalities, and telomere shortening in the pathogenesis of POI. Environmental factors seem to be the major determinants in ovarian reserve or premature menopause during the prenatal period or adult life (25). Perfluoroalkyl and polyfluoroalkyl substances (PFASs) are persistent synthetic chemicals that are widely used in industrial applications and often detectable in humans (26). Perfluorohexanesulfonate (PFHxS), perfluorononanoate (PFNA), perfluorooctanoate (PFOA), and perfluorooctanesulfonate (PFOS) are used as stain, water, and grease repellents in a wide range of consumer products. Human exposure to PFAS mainly occurs orally via the intake of contaminated food, water, and dust. A population-based study (27)showed that high exposure to PFOA, PFOS and PFHxS was linked to an increased risk of POI in humans. Jiao et al. (20)found that PFNA can induce OS, destroy spindle assembly, disrupt mitochondrial function as well as DNA damage, and cause oocyte apoptosis to interfere with oocyte maturation *in vitro*. Animal experiments have shown that exposure of mice to PFOS (0.1 mg/kg) inhibited ovarian hormone production and impaired follicular development and ovulation by selectively reducing acetylation of ovarian steroidogenic acutely regulated promoter histones, suggesting that high exposure to PFOS and PFOA may reduce ovarian follicular reserve (28). Similarly, mycotoxins, which are secondary metabolites of various fungi, have had a significant impact on food contamination in many countries, especially in developing countries. Fusarium species produce many trichothecene mycotoxins; one of them is Nivalenol (NIV). Previous studies have shown that NIV exposure in female rats disrupts the reproductive system and reduces fertility. The earlier studies (29)have demonstrated that NIV exposure in female rats disrupts the reproductive system and reduces fertility.Wang et al. found that type B Nivalenol exposure was also capable of mediating OS, leading to meiotic cell cycle arrest and further inducing failure of polar body extrusion in POI mouse oocytes, inducing autophagy and early cell apoptosis (30).

#### 2.1.1 OS-mediated DNA damage and apoptosis in POI

ROS are a group of short-lived, highly reactive, oxygenated products of OS. Their response pathway to OS-induced DNA damage has been widely demonstrated, with DNA damage and chromosome missegregation that can further lead to apoptosis (31). ROS can induce DNA damage and affect DNA damage response(DDR) by mediating genotoxin-induced damage, influencing double-strand breaks (DSBs) sensing, impacting signal transduction within DDR, interfering with cell cycle progression, and inducing p53 transcriptional response and apoptosis (32). Numerous studies have shown that ROS induces DNA breaks as well as decreased base oxidation and DNA repair in GCs, reducing the transfer of nutrients and survival factors to the oocyte, leading to apoptosis and initiating follicular atresia, which may be a key factor in the progression of POI (32–34). Apoptosis in GCs is the cellular mechanism of follicular atresia in mammals, the main process of ovarian follicle and oocyte loss, and the underlying cause of ovarian aging (35). Chemotherapeutic drugs with reproductive toxicity such as cyclophosphamide (CTX) or its main metabolite acrolein can cause excessive production of ROS *in vivo*, damage DNA structure and function in GCs, and trigger ovarian DNA repair responses and induce apoptosis in oocytes and thus leading to irreversible POI (33, 36). Sha et al. (33) and Jiao et al. (20) both demonstrated that oxidants such as H_2_O_2_ and PFNA can mediate OS leading to oxidative DNA damage and cause early apoptosis in GCs cells. ROS are capable of directly inducing DNA damage by oxidizing nucleoside bases (e.g., forming 8-oxoguanine), which can lead to G-T or G-A translocations if not repaired (37). In addition, ROS are able to oxidize deoxyribonucleoside triphosphates (dNTPs) to affect polymerase activity and reduce the rate of replication *in vitro (* 38, 39). Deltamethrin, a class of synthetic pyrethroids, is known as one of the most effective insecticides, because it kills mites and ticks by generating ROS and RNS that directly cause OS damage. Deltamethrin was found to mediate OS to attack DNA and produce genotoxicity, neurotoxicity and reproductive toxicity, including micronucleus induction, chromosomal and nuclear abnormalities and DNA breakage (40). For this reason, it is capable of causing ultrastructural changes such as substrate stripping, border folds, and irregular nuclear membranes in young oocytes (stages I-III) of ticks, interfering with oocyte development and producing reproductive toxicity (41). In addition, Deltamethrin (DM) as an insecticide is able to mediate OS to produce high levels of ROS levels, cause DSBs in DNA of oocytes and induce oocyte apoptosis by regulating Bcl-2-associated X protein (Bax) and B-cell lymphoma-2 (Bcl-2) protein expression, then reduce ovarian reserve function (42).

#### 2.1.2 OS-mediated dysfunction of mtDNA in POI

Mitochondria are a major source of ROS and they not only regulate the level of ROS produced but are also very sensitive to OS damage. ROS accumulation contributes to mitochondrial DNA damage, which further leads to mitochondrial dysfunction (43). Excessive accumulation of ROS causes damage to oocytes and GCs, which in turn leads to ovarian senescence and reduced ovarian reserve. When mitochondrial function is impaired, a mitochondrial stress response mechanism, also known as mitochondrial phagocytosis, is initiated to prevent further damage and restore mitochondrial homeostasis (9, 44). The maternal inheritance of POI and the significant dependence of folliculogenesis on mitochondrial number and energetics suggest that extensive mitochondrial defects may be involved (45). Miao C et al. (46) found that granulosa cell insufficiency in POI was associated with elevated ROS levels and reduced mitochondrial membrane potential (MMP) and adenosine triphosphate(ATP), suggesting considerable mitochondrial dysfunction. Moreover, Bakhshalizadeh S et al. found that mitochondrial ribosomal protein L50 (MRPL50) error variants destabilize mitochondrial ribosomes, leading to oxidative phosphorylation deficiency and syndrome POI (47), highlighting the importance of mitochondrial support in ovarian development and function. Mitochondria are extremely abundant in the oocyte, not only providing energy but also acting as signal transduction hubs to regulate physiological activities such as intracellular signaling, calcium homeostasis, apoptosis and autophagy, which are essential for oocyte quality (48, 49). Mitochondria produce ATP using redox reactions in the respiratory chain complex located in the cell membrane. However, a small amount of ROS is produced in mitochondria due to electron leakage in the electron transport chain (ETC). Physiologically produced ROS act as signal transducers for successful oocyte maturation and ovulation, facilitating the restoration of diploidy in the follicular microenvironment to terminate meiosis in oocytes (50). When mitochondrial dysfunction leads to excessive accumulation of ROS, nevertheless, the mitochondrial genomic DNA responsible for encoding the core components of the ETC lacks histone protection, antioxidant defense and effective DNA repair systems that are more vulnerable to damage (51). Wang et al. (52) proposed mechanisms of mitochondrial-driven ovarian aging including accumulation of mtDNA mutations, mitochondrial dysfunction, impaired fusion and fission, modified membrane potential, altered metabolism and defective ETC function, and reduced mtDNA contents. Bonomi, M et al (45). found significant mtDNA depletion by Taqman copy number assay in women with POI and poor responders to ovarian stimulation compared to 43 women of similar age with intact ovarian reserve or 53 very old women with previous physiological menopause (P < 0.001). Ding Y et al. (53) used PCR-Sanger sequencing to find that POI patients present with mutations or high variability in mitochondrial transfer Ribonucleic acid (mt-tRNA) genes, and these mutations are able to lead to impaired mitochondrial protein synthesis, significantly reduced ATP levels, and elevated ROS levels, strongly suggesting that these mt-tRNA mutations may contribute to mitochondrial dysfunction and play an active role in the progression and pathogenesis of POI. Using a zebrafish Oxr1a mutant, Xu H et al. (54)explored the role of Oxr1a in oocyte maturation and POI. oxr1a deletion leads to imbalance in the redox state of oocytes and mitochondrial dysfunction, entailing the development of a POF-like phenotype. PFNA exposure increases the level of reactive oxygen species, inducing abnormal mitochondrial distribution and increased mitochondrial membrane potential resulting in mitochondrial dysfunction in mouse oocytes (20). DM is also known to mediate OS that causes abnormal mitochondrial distribution, decreases mitochondrial membrane potential, and reduces oocyte quality (42).

#### 2.1.3 OS-mediated meiotic abnormalities in POI

OS can affect meiotic progression and also interrupt the normal assembly of meiotic structures, ultimately inducing failure of oocyte maturation and accelerating disease progression in POI (55, 56). H_2_O_2_ is a well known signaling agent and is the most direct and effective agent causing OS in cells. It is the major biological ROS, a multifunctional pleiotropic physiological signaler for many essential metabolic functions, and is often used to construct oocyte oxidative stress models and models of early-onset ovarian insufficiency. Single-cell RNA sequencing data showed that H_2_O_2_-treated oocytes caused OS mainly through two pathways: “meiosis” and “oxidative phosphorylation”. Furthermore, oxidative stress was observed in oocytes treated with H_2_O_2_ to interfere with the mitochondrial distribution pattern and membrane potential abnormalities in oocytes. H_2_O_2_ induces elevated endogenous pro-apoptosis-related molecules (Bax, Bak) and reduced anti-apoptotic molecules (Bcl-2, Bcl-xL) in ovarian granulosa cells and regulates apoptosis in ovarian sarcomeres *via* the ROS-JNK-p53 pathway (57, 58). The H_2_O_2_-driven Fenton reaction is able to disrupt spindle morphology and chromosome movement in oocytes during meiosis (59). It has been shown that high levels of ROS in oocytes cause spindle defects, polar body abnormalities and chromosomal mislocalization by acting on microtubules and causing them correctly attached to the centromere (60). Jiao et al. confirmed that PFNA exposure can mediate OS to produce reproductive toxicity in animals by inducing p-extracellular regulated kinase (ERK)1/2 mislocalization which leads to spindle abnormalities, defective aggregation of microtubule organizing center (MTOC) and chromosomal misalignment in MI mouse oocytes (20). Moreover, ROS cause DNA DSBs, block the meiotic progression of oocytes from reaching metaphase II (MII), inhibit follicle maturation and induce adverse pregnancy outcomes (56, 61). Sirtuins molecules are able to extend lifespan, regulate metabolism and repair DNA damage, and possess single ADP-ribosyltransferase or deacylase activity (62). Sirtuins molecules are able to extend lifespan, regulate metabolism and repair DNA damage, and possess single adenosine diphosphate (ADP)-ribosyltransferase (ART) or deacylase activity (62). Zhang et al. (63) found that exposure to the chemotherapeutic drug cisplatin caused high levels of ROS in mouse with POI, significantly disrupting cytoskeletal assembly which includes spindle formation and actin polymerization, thereby destroying meiotic organelle dynamics and arrangement, leading to oocyte meiotic failure and accelerating the decline of ovarian function, which was reversed by tea polyphenol supplementation to regulate ROS levels. Another study also supported the conclusion that high levels of ROS and apoptosis can disrupt the cytoskeleton and mitochondrial integrity, inhibit the meiotic process in oocytes, disrupt the structure of the cumulus cells, and reduce its fertilization potential, which can also be reversed by melatonin (MT) through inhibition of ROS levels (64).

#### 2.1.4 OS-mediated telomere shortening in POI

Telomeres are complexes of specialized repetitive DNA sequences and shelter proteins located at the ends of eukaryotic chromosomes. Its basic function is to protect linear chromosome ends from being mistaken for a broken end and being incorrectly repaired by the cell itself or undergoing processes such as DNA end joining or DNA recombination (65). As of 2009, Blackburn, Greider and Szostak were awarded the Nobel Prize for the discovery of the protective effect of telomeres and telomerase on chromosome ends. In women, the effects of reproductive aging on oocyte quality are mainly explained by telomere shortening (66). Telomeres are progressively shortened with each replication due to the inability of cell division to replicate DNA to chromosome ends, resulting in the loss of about 20-50 base pairs of per cell division. In addition, OS also accelerates telomere shortening and induces DNA damage response, impaired cell proliferation, oocyte senescence or apoptosis (17). A review that included 44 studies suggested that POI, diminished ovarian reserve and infertility are associated with shorter telomere length, hence the term telomere shortening is also called “a marker of aging” (67). High levels of ROS often lead to chromosomal instability or abnormalities, spindle defects, reduced mitochondrial function and telomere shortening in older oocytes (over 35 years of age) (68). Butts et al. observed shorter granulocyte TL and lower telomeric DNA in patients with occult ovarian insufficiency (OOI) (69), as well as in POI (70). It was shown that ROS induce local telomeric single-strand breaks (SSBs) by affecting transcription or shelterin binding of telomeric repeat-containing RNA (TERRA) at telomeres and cause collapse of replication fork and telomere loss in somatic cells (71, 72). When telomeres reach extremely short lengths, they can no longer perform their chromosomal end protection function. Since the six-membered protein complex (called shelterin) lacks sufficient binding sites at the very short telomeres, it remodels the telomeres into a “cap” structure. The loss of the telomere “cap” triggers misidentification of DNA damage response proteins at chromosome breaks, exposing and degrading chromosome ends. Robinson L.G et al. found that congenital dyskeratosis is a telomerase mutation-based disorder that may affect oocyte quality and developmental capacity through telomerase dysfunction or mutation or result in telomere shortening, ultimately leading to reduced ovarian reserve (73).

### 2.2 Alterations in OS-mediated signaling pathways of POI

**Figure 1:**
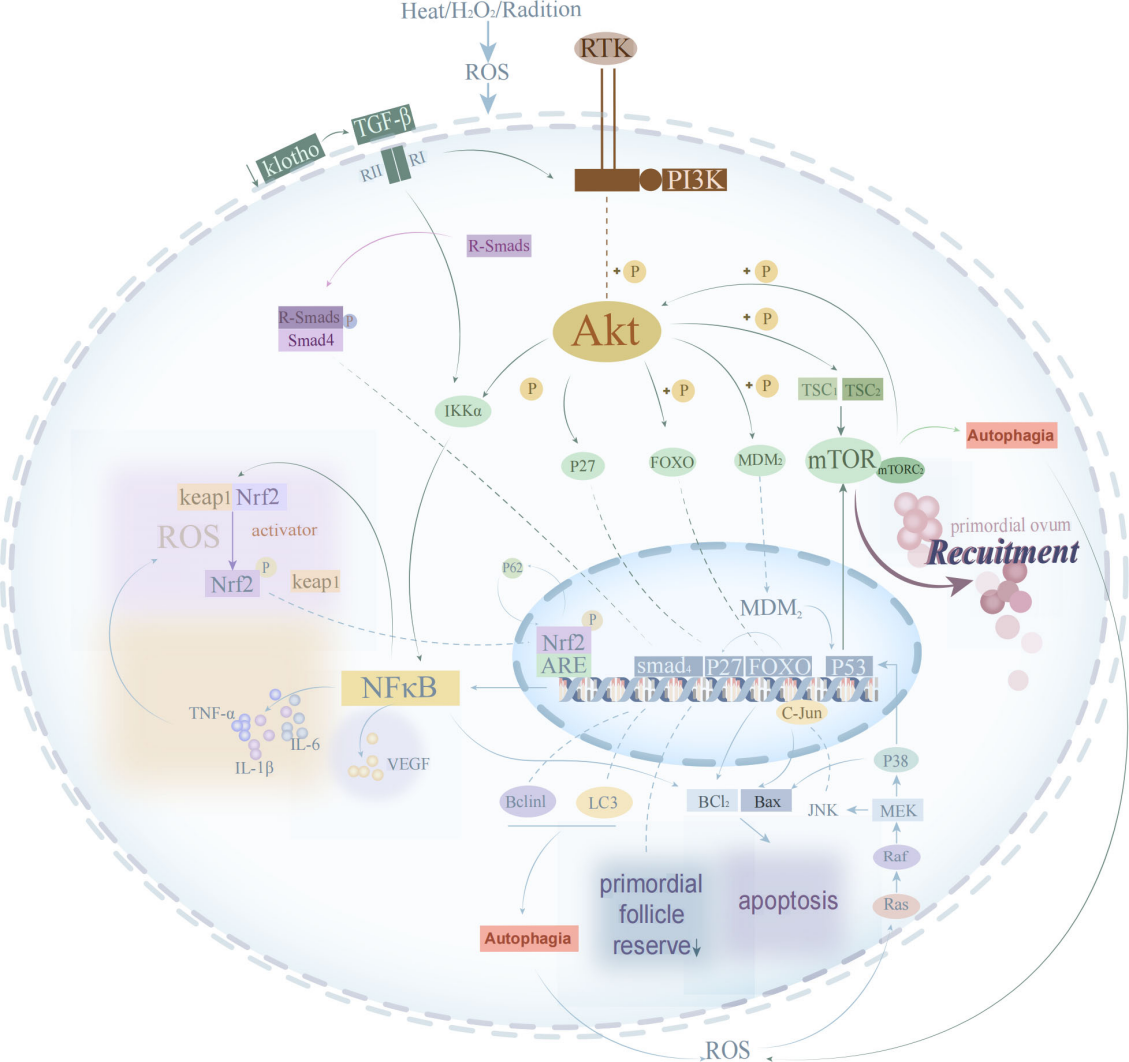
The process of oxidative stress signal transduction in oocyte.

Signaling pathways, as enzymatic response pathways, can transmit molecular signals from extracellular to cellular in order to function, and the pathogenesis of POI involves the involvement of related signaling pathways (see **Figure 1**). High levels of ROS can mediate signaling pathways such as Phosphatidylinositol 3 kinase/protein kinase B (PI3K/Akt), mitogen-activated protein kinase (MAPK), and Kelch-like ECH-associated protein 1 (Keap1)-nuclear factor erythroid 2-related factor 2 (Nrf2)-antioxidant response elements (ARE) involved in ovarian oxidative damage, GCs apoptosis, follicular atresia, and other biological processes associated with POI (74). In contrast, the transforming growth factor (TGF)-β/Smads signaling pathway can regulate cellular autophagy to reverse the manifestation of ovarian aging caused by OS.

#### 2.2.1 OS-mediated PI3K/Akt signaling pathway in POI

The PI3K/Akt signaling pathway promotes oocyte proliferation and differentiation and inhibits apoptosis (74). If abnormally blocked, the development of primordial follicles is inhibited which leads to the development of POI (75). The PI3K-Akt-mammalian target of rapamycin (mTOR) signaling pathway is competent to regulate the HPG axis (76), and the activated Akt pathway further causes a cascade of signaling pathway responses. Phosphorylated Akt can phosphorylate a series of downstream target proteins such as forkhead box type O transcription factor-3a (FOXO_3a_) (anti-proliferation and apoptosis), P_27_ (maintenance of primordial follicular reserve), Bcl-xL Bcl-2 associated death promoter (BAD) (a pro-regulatory member of the Bcl-2 family, involved in cellular mitochondrial regulatory pathways) and mTOR (controls protein biosynthesis and regulates cell growth) (74). PI3K/Akt inhibits primordial follicle overactivation by acting on the phosphorylation site of FoxO protein to avoid excessive depletion of primordial follicles and prolong ovarian reserve and fertility (77). Loss of Klotho causes attenuation of FOXO_3_ through PI3K/Akt signaling and disrupts the balance of oxidative and antioxidant systems, ultimately leading to POI (18). Quercetin (antioxidant) can restore ovarian function and inhibit OS by activating PI3K-Akt-FoxO_3a_ signaling pathway and increasing superoxide dismutase (SOD) and glutathione peroxidase (GPX) expression in POI rat (78). In the case of follicular depletion, anti-Mullerian hormone (AMH) produced by GCs of early developing ovarian follicles is also involved in protecting the primordial follicles (PMFs) pool by inhibiting FOXO_3_/FOXO_3A_ phosphorylation (leading to PMF activation) to induce ovarian autophagy (70). The PI3K/AKT/mTOR signaling pathway can regulate cell proliferation and differentiation, inhibit apoptosis by activating ribosomal kinases and promote cell cycle progression (79). Cheng Y et al. (80) found that knockdown of the TSC1/TSC2 gene was able to activate mTOR of phosphatase and tensin homology deleted on chromosome 10 (PTEN)/tuberous sclerosis complex 1 (TSC1)/tuberous sclerosis complex 2 (TSC2) in germ cells leading to primordial follicle recruitment, POI and infertility (81). Zhao Di (82) discover that the Chinese herbal medicine Erxian Decoction could promote GCs proliferation and inhibit apoptosis and exert a protective effect on cisplatin-injured ovarian GCs. On the one hand, it could promote the cell cycle transition from G0 to S phase of GCs by down-regulating the cell cycle blocking protein P_27_kip1; on the other hand, it could down-regulate the expression of FOXO_3a_ and the pro-apoptotic protein Bim.

#### 2.2.2 OS-mediated MAPK signaling pathway in POI

MAPK signaling pathway regulates apoptosis through complex mechanisms such as enhanced c-Myc expression, P_53_ phosphorylation, involvement in Fas/Fas L-mediated apoptosis, activation of c-Jun and c-fos, and induction of Bax translocation. Scholars have cloned and identified six MAPK subfamilies in mammalian cells: they are c-Jun N-terminal kinase(JNK) 1/2/3, ERK1/2, p38MAPK (p38α/β/γ/δ), ERK7/8, ERK3/4, and ERK5/BMK1 (large MAP kinase 1) (83). When different subfamilies are activated by upstream kinases, they regulate physiological processes such as inflammation, stress, cell growth, cell development, differentiation and death by phosphorylating multiple substrates such as transcription factors (84). Xu et al. (85) found that zinc pretreatment significantly reduced ROS levels in mouse embryonic osteoblasts (MC3T3-E1) via the MAPK/ERK signaling pathway and prevented OS-induced apoptosis in mouse.The JNK has been shown to induce apoptosis or growth inhibition and protect the organism from OS. Upon activation of the upstream kinases MKK4 and MKK7 (i.e. MAP2K) of the JNK pathway by different upstream MAP3Ks, respectively (83), JNKs translocate from the cytoplasm to the nucleus (86) and activate the transcription factor c-Jun translocated to the nucleus for regulating the expression of the pro- or anti-apoptotic genes Bax and Bcl-2 (87, 88). Sun et al. (89) knocked down isocitrate dehydrogenase-1 (IDH1) in a CUS mouse model and detected that elevated ROS levels in its human ovarian GCs, which induced autophagy activation and triggered cell cycle arrest in the S and G2/M phases of human ovarian GCs, which was reversed by the ROS scavenger N-acetylcysteine (NAC). Additionally, activation of ERK pathway was found to be involved in autophagy-related cell proliferation inhibition and cellular senescence, as well as JNK and p38 MAPK signaling pathways were found to be involved in regulating cell cycle arrest-related cell proliferation inhibition and senescence in IDH1 knockdown human ovarian GCs.

#### 2.2.3 OS-mediated TGF-β/Smads signaling pathway in POI

TGF-β)/Smads signaling is an important transduction pathway that regulates follicular development, and abnormalities in either process of the transduction pathway may lead to impaired signaling, resulting in impaired follicular recruitment, inhibition of follicular growth and development, accelerated follicular atresia, and POI (74). Extensive studies have shown that the transforming growth factor (TGF)-β/smad signaling pathway can regulate GCs differentiation and apoptosis, interfere with follicular development and ovulation, and is a potential mechanism for autophagy disorders in POI. Autophagy is a cytolytic metabolic pathway that helps regulate the various stages of oocyte death and follicular atresia development, and the expression or activation of autophagy-related proteins is most pronounced in high stress conditions (e.g., starvation, heat, and hypoxia) to inhibit apoptotic signals in ovarian oocytes and/or granulosa cells and promote oocyte and follicle survival, thereby ensuring fertility (90). Autophagy plays a key role in the anti-aging process through lysosomal degradation and recycling of proteins and organelles, and also plays an important part in ovarian homeostasis by reducing the oxidative load of oocytes (91). Klotho is a transmembrane enzyme that controls insulin sensitivity in organisms and inhibits OS and inflammation, whose loss leads to premature aging (62). It was indicated that reduced Klotho expression in the POI mouse model activated the TGF-β/smad signaling pathway, which in turn downregulated autophagy-related factors such as Lc3 and Beclin1 in GCs.Then, autophagy was attenuated, ROS clearance was reduced, and ROS levels increased, which subsequently led to further POI (92).

#### 2.2.4 OS-mediated Keap1-Nrf2-ARE signaling pathway in POI

The Keap1, Nrf2 and ARE signaling pathway is a classical pathway to resist endogenous and exogenous OS and a key link to inhibit the inflammatory response against endogenous and exogenous OS, which is closely associated with inflammatory diseases such as aging (93). Under physiological conditions, Keap1 targets Nrf2 for ubiquitin-dependent proteasomal degradation. During OS, Keap1 is inactivated and ubiquitination of Nrf2 is halted, leading to accumulation and activation of newly synthesized Nrf2. Thus, Nrf2 translocation to the nucleus induces the transcription of cytoprotective genes that regulate the redox state of cells to maintain cellular homeostasis (94). Nrf2 is an essential sensor and regulator of chemical homeostasis in ovarian cells that protects them from aggressive effects by managing metabolic detoxification and exerting endogenous antioxidant effects (95). In parallel with the activation of oxidative damage, Nrf2 and its cytoplasmic binding protein cooperate with Keap1 to bind to the antioxidant response element (ARE) in the target gene promoter and promote the transcription of the antioxidant gene heme oxygenase-1 (HO-1), thereby regulating the redox state of the cell to maintain cellular homeostasis (96). Chen Y et al. also verified this conclusion that ovarian tissues of POI model rats suffered from oxidative damage and the expression of Keap-1 protein was increased in ovarian tissues, while the expression of Nrf2/HO-1 protein was decreased (97). Several studies have demonstrated that Keap1-Nrf2-ARE pathway-related drugs treat age-related eye diseases through anti-OS (98). In addition, Nrf2 deficiency upregulates nuclear factor-kappa B (NF-κB) activity, leading to increased inflammatory cytokine release (99), while Nrf2 up-regulation leads to an attenuated inflammatory response in rodent models of liver and kidney injury (100). However, NF-κB activity can regulate Nrf2-mediated antioxidant gene expression. In this context, Yu et al. (93) have demonstrated that overexpression of the typical NF-κB subunit p65 increased nuclear Keap1 levels, leading to reduced Nrf2/ARE signaling. Chen (101) found that TCM activated the Keap1-Nrf2-ARE signaling pathway and downstream antioxidant enzymes SOD, heme oxygenase-1 and quinone oxidoreductase (NQO1) activities to improve ovarian function, reduce OS and inflammatory status in POI-immune model mouse. The Nrf2/HO-1 pathway also removes ROS by activating Nrf2 downstream factors, HO-1 and sequential enzymatic reactions, and the anti-senescence protein klotho inhibits ovarian and endothelial cell senescence by activating the Nrf2 pathway (102). Shang Z et al. found that excess ROS were removed by activation of HO-1, a factor downstream of Nrf2, to produce a continuous enzymatic response. The expression of Nrf2 and HO-1 decreased after D-galactose (D-gal) treatment, but increased after red ginseng extract (RGE) treatment (103).

### 2.3 OS-mediated alterations in transcription factors in POI

OS damages biomolecules and forms a series of cascade reactions that cause endogenous damage in the organism. The core of OS is the anti-apoptotic proteins (104), FOXO (105), P53 (106), Methylation of N6 adenosine/N6-methyladenosine (M^6^A) (107), and other regulatory factors that activate or inhibit signaling pathways and alter the structural and functional properties of host molecules, and transcription factor dysfunction can accelerate the onset of POI. Currently, the clinical mechanism of action for the treatment of POI includes promoting the stable expression of anti-apoptotic proteins, FOXO, P53, and exosomes, thereby reducing the damage to the ovaries caused by OS.

#### 2.3.1 OS-mediated apoptosis-related proteins in POI

Free radicals, such as ROS, have been shown to play an important role in apoptosis. Due to the uniqueness of the ovarian physiological environment and follicular development process, apoptosis of ovarian GCs and oocytes synergistically causes follicular atresia and accelerates the process of ovarian aging. The Bcl2 family is an important regulator of the endogenous pathway mediating apoptosis and can be divided into two groups: one is the regulators represented by Bcl-2 that inhibit apoptosis and suppress cell death caused by various cytotoxic factors; the other is the regulators represented by Bax that promote apoptosis and whose overexpression can antagonize the protective effect of Bcl-2 and cause cell death (108). The balance of Bax/Bcl-2 relationship can regulate apoptosis and maintain the stability of the cellular environment (109). In turn, Bcl-2-associated athanogene 3(BAG-3), as an anti-apoptotic molecular chaperone protein, also attenuates ovarian damage caused by OS. Zhu Rong et al. found that up-regulation of Bcl-2 and down-regulation of Bax inhibited follicular atresia and GCs apoptosis in POI (110). Thus, apoptosis and OS form a “vicious cycle” during POI, and therefore the stable expression of apoptosis-related proteins is important for ovarian aging (111).

##### 2.3.1.1 Bcl-2 expression inhibits apoptosis

The Bcl-2 family has a role in regulating ovarian GCs apoptosis at all stages of follicular growth and development (112).Bcl-2 is localized at sites of reactive oxygen species production such as mitochondria, ER and nuclear membranes and is a key regulator of the signals leading to activation of cysteine aspartases. Edlich F found that Bcl-2 protein prevents the release of apoptosis-associated proteins from mitochondria and resists ovarian apoptosis through aquaporin-1 protein (113). When Bcl-2 expression increases, mitochondrial membrane potential enhances and membrane permeability stabilizes, hindering the release of cytochrome C and preventing its activation of the downstream caspase family of apoptotic factors, which strengthens GCs against apoptosis and prevents the effects of OS-induced apoptosis on ovarian function (104, 114, 115). Bcl-2, despite not affecting oxygen radical production, prevents oxidative damage to GCs components, including lipid membranes (116, 117). However, it has still been suggested that Bcl-2 overexpression, although protecting primary fibroblasts from OS-mediated reduction in cell proliferation, does not prevent premature senescence (118).

##### 2.3.1.2 Increased expression of Bax accelerates apoptosis of GCs

BAX is a pro-apoptotic protein that is a key executioner of cell death by inducing permeabilization of the outer mitochondrial membrane, leading to mitochondrial regulation of cell death (119). If BAX expression increases, it causes a decrease in mitochondrial membrane potential and an increase in membrane permeability, which in turn releases cytochrome C into the cytoplasm, activates the caspase family, accelerates apoptosis of GCs, contributes to follicular atresia, and accelerates disease progression in POI (120, 121). Nevertheless, when OS-mediated ROS levels are abnormally elevated in oocytes and GCs, it causes the opening of the mitochondrial permeability transition pore, upregulates the expression of the pro-regulatory protein Bax or activates the Caspase family (122), accelerating the onset of POI.

##### 2.3.1.3 BAG-3 promotes Bcl-2 anti-apoptosis

BAG-3 is a key element of cellular proteostasis, a molecular chaperone protein that significantly promotes Bcl-2 anti-apoptosis and is involved in biological processes such as stress response, proliferation, migration and apoptosis (123). BAG3 interacts with the proteasome and regulates its activity to maintain cell survival and prolong cell life by inhibiting apoptosis for anti-aging purposes (124, 125). It was found that endothelial-specific BAG3 knockdown in mouse models significantly enhanced OS-related endothelial damage and vascular remodeling, affecting ovarian vascular function and thus accelerating ovarian aging (126). Furthermore, stress stimulation induced the expression of BAG-3, which promoted cell survival. One mechanism by which BAG3 is endowed with anti-apoptotic effects is that it binds to anti-apoptotic proteins such as κB kinase and γ inhibitors and prevents their transport to the proteasome by heat shock proteins 70 (HSP70), therefore exerting anti-apoptotic effects (127), and can attenuate the stress response. Thus, it is clear that the expression of BAG-3 can achieve its anti-apoptotic effect by inhibiting OS and attenuate the damage to ovaries by OS.

#### 2.3.2 The stable expression of FOXO can maintain normal ovarian function

FOXO is an important class of transcription factors and are important determinants of aging, whichare involved in several processes such as cell cycle arrest, metabolism and apoptosis, and DNA damage repair (128, 129). FOXO_1_ and FOXO_3_ are highly expressed in GCs of ovarian follicles, and FOXO_3a_ functions as an inhibitor of follicular activation at the earliest stages of follicular growth (130), and its sustained expression leads to retarded oocyte and follicular development and delays oocyte aging. In contrast, OS can activate the PI3K/AKT pathway and lead to inactivation of FOXO_3a_ phosphorylation, resulting in the development of POI (131). Furthermore, Foxo1-mediated changes in signaling can induce low-grade chronic inflammation *in vivo (* 132). Dabravolski S.A et al. (133) found that OS and chronic inflammation are capable of interacting and promoting each other to synergistically impair ovarian function. Beyond that, the abnormal increase in FOXO transcriptional activity can also alter the Bax/Bcl-2 ratio by regulating the expression of BH3-only protein Bim, “a direct activator of the pro-apoptotic protein Bax”, which ultimately leads to cysteine activation, DNA fragmentation and mitochondrial dysfunction, hence accelerates OS, forming a vicious cycle that speeds up the aging of oocytes (134). Cui et al. found that overexpression of FOXO aggravates OS, but deacetylation of FOXO_1_ by Silent Information Regulator 3 (SIRT3) can correct excessive OS (135), thus suggesting that stable expression of FOXO can maintain normal ovarian function.

#### 2.3.3 P53 alleviates OS and its induced oocyte senescence

P53 is a tumor suppressor gene that mediates cellular signaling pathways to regulate normal cellular life activities and intracellular redox homeostasis (136, 137). P53 is strictly regulated by the E3 ubiquitin ligase murine double minute 2(MDM2) through an autoregulatory feedback loop, and downregulation of MDM2 activity can increase the level and stability of P53, which further inhibits oocyte proliferation in order to prevent oocyte apoptosis or senescence (138). Haraguchi H et al. found that abnormal increases of P53 in ovarian GCs were able to reduce oocyte quality and ovarian function (139) and induce the appearance of premature aging phenotypes (140). Wang further discovered that OS can upregulate MDM2 and reduce P53 stability, while accelerating cellular senescence, namely, the MDM2 and P53 can form a feedback regulatory loop (141). When MDM2 is absent, however, it leads to significant P53 nuclear accumulation in oocytes, which improves the fertility potential of a mouse model resembling the human POI phenotype (142). Stable expression of P53 not only regulates oocyte quality, but also regulates oocyte autophagy. Autophagy, as a catabolic pathway, can serve as a source of energy to maintain cell cycle arrest and sustain DNA repair activities (143). Moreover, P53 is also capable of mediating cellular autophagy to remove obsolete intracellular mitochondria and alleviate OS and its induced senescence in oocytes.

#### 2.3.4 Increase in M6A impairs ovarian function

Internal modifications of messenger RNA (mRNA) are used to maintain the stability of mRNA and the most common internal modifications include M^6^A, Methylation of N1 adenosine/N1-methyladenosine (M^1^A), and 5-methylcytosine (M^5^C) (144). The M^6^A dynamic reversible modifications of RNA require methyltransferases such as methyltransferase-like 3 (METTL3) (145) and demethylases such as fat mass- and obesity-associated (FTO) (146) protein to jointly complete the regulation of mRNA function. Li Wanjing et al. (147) identified that ovarian reserve function could be improved by regulating ovarian RNA-M^6^A methylation modification, and another study confirmed that the M^6^A content in the RNA of POI patients and mice was significantly higher than that of the control group (148). Downregulation of M^6^A demethylases including FTO protein and ALKB homolog 5 (ALKBH5) leads to increased M^6^A in aged ovarian GCs, while knockdown of GCs in FTO shows a more rapid ovarian premature aging phenotype (149), thus increased M6A impairs ovarian function and accelerates the process of ovarian aging. Nrf2 is an endogenous antioxidant pathway, and it has been found that FTO can increase Nrf2 expression by mediating M6A demethylation of Nrf2 mRNA, thereby inhibiting OS (150), which shows that a decrease in M6A concentration is beneficial in alleviating OS. Additionally, M^6^A modifications can regulate cellular ROS levels through different mechanisms, and imbalanced OS and antioxidant imbalance can also increase M6A levels (107). It is thus hypothesized that OS causes an increase in M6A in mRNA, impairing ovarian function and accelerating the process of POI, but further confirmation is needed.

#### 2.3.5 Sirtuin mediate the OS involved in ovarian aging

The sirtuins are an evolutionarily conserved family of nicotinamide adenine dinucleotide (NAD)-dependent histone deacetylases that consist of seven members including SIRT1-7, which are involved in many biological processes such as cell survival, senescence, proliferation, and apoptosis by regulating inflammation, OS, and mitochondrial function (151, 152). Zhang et al. found a positive correlation between sirtuins and treated with chemotherapy mouse models (153). Another study showed that SIRT1 deficiency significantly increased ROS and inflammation levels, and SIRT3 can promote mitochondrial oxidative metabolism in response to nutritional stress and membrane depolarization to attenuate OS (154), thus it can be assumed that sirtuins can mediate OS involved in ovarian aging. Sirtuins-mediated OS and inflammation also lead to the expression of genes like Nrf2, NF-kB, interleukin (IL)-1, FOXO and P53, which also damage oocytes (155). SIRT2 and SIRT3 are able to positively regulate FOXO_3_ and increase ovarian reserve; conversely, defective sirtuins expression reduces follicle number and accelerates the depletion of PMFs (156). Yang, M., et al. found that increased expression of SIRT1, SIRT2, and SIRT3 reduced P53 acetylation levels, inhibited P53 hyperactivation, and diminished follicular apoptosis and atresia. Meanwhile, the upregulation of sirtuins inhibited P53-dependent cell cycle arrest and apoptosis (157), reducing abnormal follicular atresia to maintain normal ovarian function (158, 159).

### 2.4 OS-mediated changes in ovarian microenvironment in POI

The ovarian microenvironment refers to the environment of cell survival in which various intercellular and humoral components of the ovary participate together to constitute. It contains rich microenvironmental signals such as gap junctions, paracrine, autocrine, endocrine and exocrine signaling factors that facilitate intercellular communication. Oxidative damage to biomolecules caused by OS can lead to the production and release of cytokines through initiating endogenous damage related molecular patterns in the organism. Cytokines further recognize receptors and activate downstream signaling pathways, such as nuclear factor kB (NF-kB), janus kinase (JAK), signal transducer and activator of transcription (STAT) and MAPK, thus providing increased release of cytokines and inflammatory factors, recruiting and activating more inflammatory cells and causing a chronic inflammatory response in the body. ROS are oxygen-containing reactive chemicals that are involved in cell signaling and promote cell survival, proliferation, and differentiation at a physiological level (160). Once ROS levels exceed cellular antioxidant levels, they react with DNA, proteins, lipids and carbohydrates; leading to DNA strand breaks as well as protein and lipid oxidation. Not only do they impair ovarian function and accelerate the onset of premature ovarian failure (161–163).

#### 2.4.1 Low-level chronic inflammatory state in POI

Existing studies have implicated that OS is of great importance in the development and perpetuation of inflammation, increasing the levels of inflammatory factors such as tumor necrosis factor-α (TNF-α), IL-1β, IL-6, and TGF-β *in vivo (* 164). Han et al. (165) detected that elevated levels of IL-4, IL-1β, and IL-6 in follicular fluid samples and GCs from POI patients, and further found that IL-4 could activate the PI3K/AKT signaling pathway, promote apoptosis, and inhibit GCs proliferation in *in vitro* experiments. Besides, inflammatory factors can also activate NF-κB signaling pathway and affect the expression of follicle-stimulating hormone receptor(FSHR) through glucose-regulated protein 78-mediated ER stress to promote GCs proliferation. Luo et al. (158) noticed that decreased nuclear p65 expression in ovarian GCs and reduced number of sinus follicles in a mouse model of NF-κB inactivation, showing that OS can increase the concentration of inflammatory factors and further decline the expression of NF-κB to participate in the POI process. However, over-activation of NF-κB can induce the expression of cytokines such as IL- 1β, adhesion molecules, immune receptors, and inflammation-related enzymes, which aggravate the damage to the ovary by OS and thus form a vicious circle, thereby suggesting that stable expression of inflammatory factors is beneficial for the maintenance of ovarian function. Mantawy EM et al. (166) discovered that salicin was able to ameliorate radiation-induced POI by decreasing the expression of radiation-induced ovarian damage markers including NF-κB, TNF-α, inducible nitric oxide synthase (iNOS) and cyclooxygenase (COX)-2 and down-regulating the TGF-β/MAPK signaling pathway to impede POI-related inflammatory and apoptotic signaling pathways. We found in another study that in a fluorosis POI model, inflammatory factors IL-6 and IL-8 were significantly increased, ovarian ultrastructure was obviously damaged and showed premature failure, and there was a positive correlation with the dose of fluoride dosing, which indicated that the concentration of pro-inflammatory factors was positively correlated with the degree of POI (167). While some potential drugs for the treatment of POI can reduce the levels of inflammatory factors and modulate the ovarian microenvironment thus exerting a therapeutic effect on POI For example, resveratrol can lead to a significant decrease in TNF-α and IL-6 concentrations in the mice’s ovary (159), lacrimalin down-regulates peripheral blood CC chemokine receptor 9 (CCR9), C-X-C-motif chemokine receptor 3 (CXCR3) and CD4 T lymphocyte counts and IL-12 secretion (168).

#### 2.4.2 OS-mediated peroxidation of protein and lipid in POI

##### 2.4.2.1 Lipid peroxidation in POI

Lipids maintain the structural integrity of cells. Lipids containing polyunsaturated fatty acids are susceptible to oxidation triggered by free radicals, causing chain reactions to form lipid peroxidation products such as malonaldehyde and 4-hydroxynonenoic acid, which alter the fluidity and permeability of cell membranes and cause cellular dysfunction in aging and most age-related diseases. Disruption of redox homeostasis is a key phenotype of POI (169–172) and is closely associated with lipid peroxidation. Among the lipid oxidation metabolites, arachidonic acid metabolism is dramatically altered. Arachidonic acid may induce apoptosis by attenuating oocyte proliferation, migration and viability in response to mitochondrial membrane depolarization and activation of cystathione-3, -8 and -9 (173–175). 4-Hydroxynonenoic acid disrupts signal transduction and protein activity, as well as induces chronic inflammation and triggers apoptosis in the ovary under conditions of OS (176), contributing to decreased reserve function and ovarian aging.

##### 2.4.2.2 Protein peroxidation in POI

The accumulation of oxidatively modified proteins is a signature of aging of organisms *in vivo* and cellular replicative senescence *in vitro (* 177). Protein carbonylation is one of the most deleterious irreversible oxidative protein modifications and is the initiating factor for mitochondrial dysfunction and ER stress (178), which is also considered to be a major marker of OS-related diseases (179), with elevated concentrations in senescent cells (180). One research identified by mass spectrometry that almost half of the carbonylated proteins are located in mitochondria, reflecting the preferential accumulation of damaged proteins in mitochondria during cellular senescence (181), and impaired mitochondrial function is capable of inducing OS responses. In a research (182), PFOA was found to considerably increase the production of ROS, protein carbonylation and DNA damage in oocytes, impairing the maturation and viability of porcine oocytes and affecting their fertility. Protein damage leading to carbonyl formation originates from the direct oxidation of amino acid side chains, but can also be obtained through the formation of protein adducts of lipid peroxidation products and dicarbonyl glycation compounds (183). Under OS conditions, consequently, protein peroxidation and lipid peroxidation interact and promote each other, speeding up the occurrence of POI.

#### 2.4.3 OS-mediated impaired angiogenesis in POI

Several factors that promote and inhibit angiogenesis around the follicle work together to regulate ovarian angiogenesis, and vascular endothelial growth factor (VEGF) plays a key role in ovarian angiogenesis, especially during follicular development and ovulation (184). Selection and ovulation of the dominant follicle depends on a well-functioning vascular supply and permeability within the antral follicle, maintaining the normal function of the ovary accordingly. Instead, damage to the endothelium and inadequate capillary network formation in the membrane cell layer can lead to follicular atresia or degeneration (185, 186). Insufficient blood supply leads to a hypoxic state in the follicular fluid and inhibition of angiogenesis which can cause disruption of follicular development, ovulation and ovarian endocrine function (187). There is also some research showing that ovarian tissue VEGF protein expression is clearly lower in rats with POI, demonstrating that follicle development is closely linked to angiogenesis (188). Mitochondrial ROS is instrumental in promoting angiogenic conversion in resting endothelial cells (189), and OS impairs lysosomal clearance of damaged DNA, enhances stimulator of interferon genes (STING) signaling, and up-regulates VEGF expression in senescent cells, which is beneficial for angiogenesis (190). Hence, good regulation of OS promotes angiogenesis and tissue repair (191), stimulates ovarian endothelial cell motility and proliferation *in vitro* or vivo, boosts follicle development, and reduces apoptosis (192). Nonetheless, vascular endothelial dysfunction occurs when the increase of immune cells in the vessel wall leads to the release of inflammatory cytokines and induces the production of ROS, which eventually leads to the generation of excessive OS (193), entailing a disruption of vascular homeostasis. OS-mediated epithelial cell injury may also contribute to a decrease in VEGF levels (194), and it has been evidenced that young women with POI have evident vascular endothelial dysfunction (195), so maintaining OS at physiological level is beneficial for the stability of the ovarian microenvironment and slows down the process of ovarian aging.

#### 2.4.4 OS-mediated exosomes in POI

Exosomes are small membrane vesicles (30-150 nm) containing complex RNAs and proteins, which are crucial for intercellular communication and information transfer and are able to improve POI by inhibiting apoptosis and promoting angiogenesis (196, 197). Some of these healthy cells, including MSCs, neural progenitor cells and astrocytes, have antioxidant damage and their derived exosomes show the same efficacy as parental cells (198, 199). It was shown that miR-144-5 p carried by bone marrow mesenchymal stem cell-derived exosomes inhibited the expression of apoptosis-related proteins by suppressing the PTEN, thereby up-regulating the PI3K/ATK pathway to promote ovarian follicle recovery after chemotherapy and improving follicle morphology in POI mouse. This conclusion was verified by Yang, Meiling et al. (200), Exosomes inhibited cisplatin-induced GCs apoptosis and increased cell viability. In contrast, OS can notably affect the number of exosomes by regulating the degradation of multivesicular bodies. High levels of OS promote the degradation of intracellular multivesicular bodies by activating autophagy, lessening the amount of exosomes, thereby influencing exosome function, reducing the antioxidant effect of exosomes, and exacerbating the damage of OS on oocytes (201). Qu et al. determined that human umbilical cord MSC-derived exosomes carrying miR-126-3p promoted angiogenesis in POI rats and attenuated apoptosis of ovarian GCs, highlighting the potential of miR-126-3p-containing exosomes as an effective therapeutic strategy for POI treatment (202). In addition, Sun et al. (203) discovered that miR-644-5p carried by bone mesenchymal stem cell-derived exosomes could inhibit apoptosis of ovarian GCs by targeting cellular p53, suggesting the potential of exosome-carried micro Ribose Nucleic Acid(miRNA) to treat POI and restore ovarian function through their antioxidant effects.

## 3 The application of antioxidants in POI

The key to counteracting ovarian functional impairment in POI patients caused by OS is to restore the dynamic balance between ROS and the antioxidant system by reducing the production of ROS on the one hand and increasing the activity of the endogenous antioxidant system or supplementing exogenous antioxidant substances to enhance the ability to scavenge ROS on the other hand (204). The endogenous antioxidant system of the organism consists of two main components, an enzymatic scavenging system consisting of various antioxidant enzymes such as SOD and catalase (CAT), and a non-enzymatic scavenging system comprising antioxidants including GSH and MT (205). Furthermore, herbal extracts such as ginsenosides, curcumin (CUR), resveratrol and stem cell transplantation also perform important roles in the fight against OS, and we will introduce several antioxidants with outstanding efficacy and good research prospects (**Table 1**).

**Table 1:** The application of antioxidants.

Category	Reference	Design	Model	Sample size	Intervention	Outcomes
Antioxidant	Zhang, H., et al., Melatonin improves the quality of maternally aged oocytes by maintaining intercellular communication and antioxidant metabolite supply. Redox biology, 2022. 49: p. 102215.	randomised controlled trial (RCT)	mouse	105	Yong group: not processed Old group: water Old+MT group: melatonin	*In vitro* MT therapy can fight OS by maintaining glucose-6-phosphate dehydrogenase (G6PDH) activity, nicotinamide adenine dinucleotide phosphate (NADPH) and GSH levels in the cumulus oocyte complex (COC) of the elderly. In the absence of cumulus cells, MT can reduce ROS level in oocytes.
	Xu, H., et al., Melatonin Protects Against Cyclophosphamide-induced Premature Ovarian Failure in Rats. Human & Experimental Toxicology, 2022. 41: p. 09603271221127430.	RCT	Rat	48	Control group: 0.9% normal saline POI group: CTX MT10 group: 10 mg/kg MT+CTX MT20 group: 20 mg/kg MT+CTX	Compared with POI group, MT group: FSH ↓, luteinizing hormone (LH) ↓ (p<0.05), P ↑, E2 ↑, AMH ↑ (p<0.01). The number of follicles in MT treatment group was significantly higher than that in POI group.
	Delkhosh, A., et al., Upregulation of FSHR and PCNA by administration of coenzyme Q10 on cyclophosphamide‐induced premature ovarian failure in a mouse model. Journal of Biochemical and Molecular Toxicology, 2019. 33(11): p. e22398.	RCT	mouse	42	12 male rats: *in vitro* fertilization 32 female mice: Health control group: sesame oil CTX group: CTX CTX+CoQ10 group: CTX+CoQ10 CoQ10 group: CoQ10	Compared with CTX treatment group, after CoQ10 treatment, the abnormal rate of ovarian morphology and structure was significantly reduced, the loss of follicles was prevented, the number of atresia follicles was reduced (P<0.05), and the ROS level in MII oocytes was significantly reduced (P<0.05)
	Mantawy, E.M., et al., Novel molecular mechanisms underlying the ameliorative effect of N-acetyl-L-cysteine against ϒ-radiation-induced premature ovarian failure in rats. Ecotoxicology and Environmental Safety, 2020. 206: p. 111190.	RCT	Rat	60	Control group: normal saline Irradiation group: normal saline+irradiation NAC group: NAC NAC/irradiation group: NAC+irradiation	NAC treatment reduced E2 by 73%, AMH by 40%, GPX activity by 102%, p22 by 48%, NADPH oxidases 4 (NOX4) by 38%, p53 33%, Bax16%, Bcl-2 mRNA expression by 135%, caspase3 activity by 43%, and VEGF expression by 43%
	Jiang, Y., et al., Resveratrol plays a protective role against premature ovarian failure and prompts female germline stem cell survival. International Journal of Molecular Sciences, 2019. 20(14): p. 3605.	RCT	mouse	Not mentioned	RES low concentration group (20 mg/kg): 20 mg/kg RES RES high concentration group (40 mg/kg): 40 mg/kg RES POI group: cyclophosphamide +Busulfan induction Control group: untreated (the control group was not found in the original text)	Mouse weight ↑ (p<0.001), ovarian weight ↑, follicular atresia rate ↓, mouse vasa homologue (Mvh) ↑, octamer-binding transcription factor 4 (Oct4) ↑ (p<0.001 or p<0.01), SOD2 ↑, GPX ↑, CAT ↑, tumor necrosis factor-α (TNF- α) ↓, IL-6 ↓, IL-10 ↑ (p<0.05, p<0.01 or p<0.001), malondialdehyde (MDA) ↓, FGSC survival rate ↑ (p<0.001 or p<0.01), ratio of primary follicles to primary follicles ↑ (p<0.001 or p<0.01)
	Yan Qian et al., Protective effect of ginsenoside Rg_1 on cisplatin-injured rat ovary granule cells and its molecular mechanism. Zhongnan Pharmacy, 2022. 20(05): p. 1028-1033.	RCT	Rat	16	Normal group: untreated Model group: cisplatin Low concentration group of ginsenoside Rg1: cisplatin+low concentration ginsenoside Rg1 Ginsenoside Rg1 medium concentration group: cis Platinum+medium concentration ginsenoside Rg1 Ginsenoside Rg1 high concentration group: cisplatin+high concentration ginsenoside Rg1 E2 group: cisplatin+ β- Estradiol	Compared with the model group, after treatment with ginsenoside Rg1, the survival rate of ovarian GCs ↑, the decrease of cell proliferation rate was significantly inhibited, FSHR ↑, PI3K ↑, p-AKT/AKT ↑, Bcl-2/Bax ↑ (P<0.05)
	Yanjun Han, Mechanism of ginsenoside Rg1 induced aging in human leukemia cells (K562) based on PI3K/Akt/mTOR autophagy pathway, 2020, Dali University.	RCT	Human chronic myelogenous leukemia K562 cells	54	D-gal group: D-gal, continuous administration for 42 days PBS group: equal amount of phosphate buffer solution (PBS), continuously injected for 42 days Rg1 group: on the basis of D-gal group treatment, Rg1 was injected intraperitoneally on the 15th day, and was continuously administered for 28 days	Compared with D-gal group, Rg1 group: ovarian SA- β- Positive rate of galactosidase staining ↓, P16 INhibitorofcyclin—dependentKinase4a (P16INK4a)↓ (P<0.05), Microtubule-associated protein light chain (LC3 - II) ↑ (P<0.05), PI3K ↓, Akt ↓, mTOR ↓, S6k ↓ (P<0.05), Akt mRNA ↓, mTOR mRNA ↓, S6k mRNA ↓ (P<0.05)
	Zhengjie Yan, Curcumin in delaying premature ovarian failure and its mechanism, 2021, Yangzhou University.	RCT	mouse	60	D-gal molding group: D-gal CUR treatment group: D-gal+CUR Control group: physiological saline with the same volume as D-gal	Compared with D-gal model group, CUR treatment group: p-Akt ↑ (P<0.01), caspase-3 ↓, caspase-9 ↓ (P<0.01), Nrf2 ↑ (P<0.05), HO-1 ↑ (P<0.01)
mesenchymal stem cell	Huang, B., et al., Fetal liver mesenchymal stem cells restore ovarian function in POI by targeting MT1. Stem cell research & therapy, 2019. 10(1): p. 1-12.	RCT	mouse	150	Normal group: not treated POI group: single intraperitoneal injection of cyclophosphamide fMSC group: received tail vein injection of about 1 × 106 fMSC 2 weeks after CTX injection	Compared with POI group, fMSC group: number of sinus follicles ↑, total number of follicles ↑, E2 ↑, AMH ↑ (p<0.001), ROS rate (16.3%) ↓, MDA ↓, lactate dehydrogenase (LDH) ↓, SOD ↑, glutathione reductase (GR) ↑, CAT ↑, GPX ↑, *in vitro* and *in vivo* melatonin membrane receptor 1 (MT1) ↑, JNK1 ↑, Proliferation cell nuclear antigen (PCNA) ↑, AMP-activated protein kinase (AMPK) ↑
	Ding, C., et al., Exosomal miRNA-17-5p derived from human umbilical cord mesenchymal stem cells improves ovarian function in premature ovarian insufficiency by regulating SIRT7. Stem Cells, 2020. 38(9): p. 1137-1148	RCT	mouse	40	Wild type (WT) group: no treatment POI group: CTX Exos/POI group: secretions from CTX+hUMSCs Exosanti-miR-17-5p/POI group: secretions from hUMSCs knocked down by CTX+miR-17-5P	human umbilical cord mesenchymal stem cells (hUMSC) - Exos restored the ovarian phenotype and function of the POI mouse model by delivering miR-17-5P and inhibiting the expression of SIRT7, promoted the proliferation of human granulosa cells (hGCs) and ovarian cells damaged by CTX, and reduced the accumulation of ROS.
Biological Enzymes	Tang, X., et al., Ubiquitin-like modifier 1 ligating enzyme 1 relieves cisplatin-induced premature ovarian failure by reducing endoplasmic reticulum stress in granulosa cells. Reproductive Biology and Endocrinology, 2022. 20(1): p. 1-16.	RCT	mouse	Not mentioned	Control group: not treated Mouse POI model group: construction of cell culture *in vitro* with cisplatin: treatment of primary granular cells with cisplatin knockdown (KD)-1+Cis and KD-2+Cis: Sh RNA lentivirus transfection particles knock down UFL1	Mouse POI model group: UFL1 specific ligase 1 ↓, ER stress ↓, FSHR ↓ Compared with the control group, the UFL1 knockdown group: the total number of normal ovarian follicles ↓, the comparison of original follicles ↓, the proportion of atresia follicles ↑, and the levels of FSHR ↓, AMH ↓ and E2 ↓ in ovarian tissue. Compared with POI group, FSHR ↓, AMH ↓, apoptosis-related molecular protein Bax/Bcl-2 ↑, and the ratio of cleav ed caspase-3/caspase-3 ↑ in KD-1+Cis and KD-2+Cis groups

A randomized controlled trial is a means of testing the effects of a therapy or drug in health care. The basic method of randomized controlled trials is to randomly group study subjects and implement different interventions for different groups to control the differences in effects. It has many advantages such as being able to minimize various possible biases in the design and implementation of clinical trials, balance confounding factors, and improve the validity of statistical tests, and is recognized as the gold standard for evaluating interventions.

### 3.1 The application of antioxidant in POI

#### 3.1.1 The application of melatonin in POI

MT, an amine hormone produced by the pineal gland in mammals and humans, is secreted in tissues such as the ovary and placenta (206). In the long-term decline of ovarian aging, melatonin treatment is able to regulate ovarian function through anti-oxidative stress and also protect the follicular pool, oocyte quantity and quality (207). Multiple studies have indicated that MT protects mice’ and hamsters’ GCs and oocytes from ROS damage by reducing OS (208, 209), thereby protecting ovarian function and delaying the development of POI. Osatd-Rahimi N et al. (210) found that melatonin increases the activity of CAT, SOD and thiols, thus it has a protective effect against oxidative stress. Additionally, melatonin plays an effective role in reducing malondialdehyde (MDA) levels, which are a product of lipid peroxidation in cell membranes. It can directly scavenge reactive oxygen radicals, promote the synthesis of the reactive oxygen scavenger GSH in the body, and mitigate OS damage to mitochondrial proteins and DNA (211). Cyclophosphamide (CTX), an alkylating agent with the highest risk of ovarian damage, is commonly used in the treatment of malignancies such as breast cancer (212). According to studies, CTX is catalyzed and degraded *in vivo* by hepatic microsomal enzymes, resulting in the production of phosphoramidite mustard and acrylic acid, which have cytotoxic effects (213). During the course of treatment, abnormal blood circulation and associated stress damage to the body may result in the release of large amounts of free radicals. It promotes peroxidation and releases large amounts of ROS, which damages the mitochondrial and cellular membranes of ovarian granulosa cells, induces an inflammatory cascade, releases alarm signals (alarms), and initiates cellular responses (214). Whereas granulosa cells in the ovary provide an important environment for oocyte formation and development (215). CTX alters follicular growth, oocyte maturation and apoptosis or atresia in the ovary due to the production of ROS and inflammatory substances. Xu et al. (216) examined that MT improved POI in a cyclophosphamide-induced POI in rat model by maintaining ovarian hormonal status, enhancing ovarian index, promoting follicular development, and inhibiting GCs apoptosis. Animal and human trials showed that short-term use of MT was safe and long-term MT treatment caused only minor adverse effects comparable to placebo (217).

#### 3.1.2 The application of sphingosine 1-phosphate in POI

Sphingosine-1-phosphate (S1P) is a biologically active sphingolipid that mediates various biological processes such as apoptosis, immune response and inflammation, and can substantially reduce oocyte apoptosis initiated by various stimuli such as radiation and chemotherapeutic drugs, effectively protecting ovarian tissue to alleviate POI (218). It was found that OS can induce cellular regulation by altering the balance of Cer/S1P, and with elevated Ceramide (Cer)/sphingosine-1-phosphate (S1P) levels, cells tend to move in a direction favorable to life activity. Conversely, cells tend to die (219), so S1P is an important antioxidant in reducing apoptosis, and thus treating POI. There are fewer reports on its safety, and one study detected that S1P treatment interferes with the clinical effects of anticancer drugs during chemotherapy, and its anti-apoptotic effects may inhibit normal atresia of DNA-damaged oocytes during follicular development (206).

#### 3.1.3 The application of coenzyme Q10 in POI

Coenzyme Q10 (CoQ10) is a fat-soluble quinone, a natural antioxidant found in the mitochondrial respiratory chain. Exogenous supplementation of CoQ10 has been shown to improve ovarian function in mouse (220). It has a beneficial effect on POI patients by reducing OS, preventing mtDNA mutations caused by peroxidative damage, restoring mitochondrial function, counteracting ovarian physiological aging and improving ovarian reserve function for enhancing ovarian responsiveness (221). Delkhosh A et al. (220) discovered that CoQ10 markedly improved ovarian histological morphology and the number of atretic follicles in CTX-treated mouse ovaries by down-regulating ROS levels. It was also shown that CoQ10 is well tolerated, has few adverse effects, and is highly safe (222).

#### 3.1.4 The application of N-Acetyl-L-cysteine in POI

N-Acetyl-L-cysteine (NAC) is a thiol antioxidant whose main role is to disturb the generation of free radicals and to scavenge those already generated (223). Mantawy EM et al. (224) revealed that NAC inhibits radiotherapy-induced POI by down-regulating nicotinamide adenine dinucleotide phosphate oxidase (NOX) and p22 proteins and enhancing antioxidant defense to eliminate oxidative damage, and suppresses p53-dependent apoptotic mechanisms by reducing multiple pathways including MAPK signaling pathway, and also reduces follicular atresia and increases ovarian reserve by inhibiting radiation-triggered primordial follicular loss, thereby restraining radiotherapy-induced POI in rats. At the same time, it preserves ovarian function and structure, and plays a positive role in POI patients. Clinical studies have proven that NAC has a good safety and tolerability profile (225).

#### 3.1.5 The application of natural compounds and herbal extracts in POI

Vitamin C and E are classic antioxidant molecules. Vitamin C converts oxidized glutathione to reduced glutathione. The α-tocopherol in vitamin E can directly scavenge excess ROS from the body, modulate antioxidant responses, and enhance the expression of antioxidant enzymes like SOD (226), thus exerting an antioxidant stress effect and favorably affecting POI patients. Resveratrol (RES), a naturally occurring dietary antioxidant flavonoid, is the most recognized antioxidant and the strongest activator of the longevity gene SIRT1. It can effectively improve ovarian function and female germline stem cell (FGSC) regeneration in POI models by alleviating OS, inflammation, and mechanisms involving the Hh signaling pathway (159), while FGSCs can initiate meiosis in the ovary to produce oocytes and expand follicular reserve to preserve fertility. RES also apparently rescues the activity of antioxidant enzymes including GPX, SOD, and CAT in POI ovaries (159) and counteracts OS, which plays an essential role in the treatment of POI. The ginseng extract ginsenoside Rg1 has a protective effect on ovarian function and can delay ovarian aging through its antioxidant, anti-aging, anti-inflammatory and estrogenic activities (227). Rg1, a ginseng extract, not only can inhibit GCs apoptosis and promote the proliferation and growth of GCs by up-regulating the expression of FSHR and activating the FSHR-PI3K-AKT pathway to protect ovarian function in rat model (228) and thus attenuate POI. D-gal was able to successfully induce POF models by impairing follicular development, decreasing the apoptosis rate of granulosa cells, lowering E2 levels, and increasing the level of FSH *in vivo*. Studies have shown that D-gal can induce oxidative stress directly in the body, and that increased levels of galactose in women’s blood lead to a decrease in the number of ovarian follicles and damage to oocytes and ovarian granulosa cells, thus leading to POI (229, 230). Rg1 can also can postpone D-galactose (D-gal)-induced POI through the PI3K-Akt-mTOR autophagy signaling pathway mouse model of ovarian senescence, effectively facilitating the secretion of estrogen and luteinizing hormone, and increasing the expression of SOD and CAT, thereby alleviating ovarian oxidative damage in mice model (231, 232) and delaying the development of POI. Aside from this, Tang’s rat experiments demonstrated that ginsenoside Rb1 has a protective effect on ovarian function by the mechanism that the estrogenic activity of ginsenoside can regulate ovarian function, reverse disorders in sex hormone levels, reduce apoptosis of ovarian granulosa cells, and delay ovarian aging (233). We have discovered that CUR (the active ingredient of turmeric rhizome) possesses a positive effect on POI patients by potently inhibiting D-gal-induced 0S, apoptosis and ovarian damage through various mechanisms including Nrf2/HO-1 and PI3K/Akt signaling pathways (234) (193). Compared with resveratrol and vitamin C, the antioxidant capacity of CUR is superior (234).

Relevant animal and clinical studies have confirmed that both the above natural compounds and herbal extracts have a good safety profile in animal models and human models (235–239).

### 3.2 The application of mesenchymal stem cells in POI

Numerous studies have recently reported promising therapeutic effects of MSC for POI treatment (240) (241–243). MSCs can secrete a variety of factors, including cytokines and exosomes, that lead to POI recovery through several mechanisms, involving reduction of apoptosis and inflammation and induction of angiogenesis (244). Stem cell transplantation is a treatment that has the ability to repair damaged ovarian tissues, restore ovarian elasticity, and reduce ovarian tissue fibrosis. In recent years, MSCs for the treatment of POI have become a hot research topic in this field. The mechanisms include MSC homing, promotion of cell proliferation, inhibition of apoptosis, differentiation, anti-inflammatory and immunomodulation, secretion of various cytokines, regulation of genes, and regulation of cell autophagy (245). The main MSCs used for the treatment of POI are bone marrow mesenchymal stem cells, adipose mesenchymal stem cells (ADMSCs), placental mesenchymal stem cells (PMSCs), amniotic mesenchymal stem cells (AMSCs), umbilical cord mesenchymal stem cells (UCMSCs), amniotic fluid mesenchymal stem cells (AFMSCs), and menstrual stem cells (MenSCs). We have discovered that MSCs can play an anti-OS role by secreting IL-6, VEGF and other related cytokines and exosomes, activating NQO1/MAPK and other related pathways, increasing the production of antioxidant-acting enzymes, and prohibiting ROS production, which in turn improves mice’ ovarian function (246). For example, fetal mesenchymal stem cells (fMSCs) prominently reduce oxidative damage, increase oxidative protection, improve anti-apoptotic effects, and inhibit apoptotic gene expression *in vivo* and vitro (247), protecting ovarian function in POI patients. Exosomes have been recognized to have a vital role in the treatment of POI by MSC, which are carriers of intercellular signaling and drug therapy, and miRNA is a class of single-stranded non-coding RNA in exosomes. Several studies have found that human mesenchymal stem cells derived from bone marrow (BMSC)is at work in the fight against oxidative stress. Proteomic analysis revealed that various antioxidant mediators secreted by BMSCs, such as cyclophilin A, cyclophilin B, thioxapurine, DJ-1, heat shock protein 27, and peroxycin-1, exhibited significant antioxidant stress effects. Proteomic analysis revealed that various antioxidant mediators secreted by BMSCs, such as cyclophilin A, cyclophilin B, thioxapurine, DJ-1, heat shock protein 27, and peroxycin-1, exhibited significant antioxidant stress effects (248). Insulin-like growth factor 1 (IGF1) released from BMSCs also has potent antioxidant capacity (249). On top of that, BMSC transplantation affects SOD activity and malondialdehyde content by decreasing cyclin-dependent kinase inhibitor 2A(P16) expression and increasing proliferating cell nuclear antigen, so as to improve the morphology and function of the ovary (250). Exosomes and miRNAs have the capacity to regulate the expression of downstream target genes to inhibit apoptosis of various cells in the ovary, so as to improve ovarian function (251) and slow down the progression of POI. For instance, human umbilical cord MSC-derived exosome microRNA-17-5P (miR-17-5p) can reduce ROS accumulation by inhibiting SIRT7 expression to combat OS and thus improve POI (252).

Although MSC has obvious therapeutic effects in animal models of POI, its clinical application still suffers from the issues of insufficient cell source, immunogenicity, passaging culture, ethics, safety and efficacy, which need to be evaluated more comprehensively through more in-depth clinical studies (245, 253).

### 3.3 The application of bioenzymes in POI

Endogenous antioxidant systems exist within the body and cells, including antioxidant enzymes such as SOD, GPX, glutathione sulfhydryltransferase (GST), and CAT, in addition to GSH as a scavenger of ROS (205, 223). Superoxide anions in ROS form a spontaneous conversion to H_2_O_2_ catalyzed by SOD, and then CAT and GPX further convert H_2_O_2_ to O_2_ and H_2_O, which exerts antioxidant stress effects in POI patients (254). In addition, phase II detoxification enzymes have defensive effects against oxidative damage caused by exogenous chemical exposure, mainly including HO-1, HQO1 and γ-glutamylcysteine ligase (γ-GCL), which can moderate POI to some extent by counteracting OS. HO-1 is a classical phase II detoxifying enzyme involved in preventing the generation of more active hydroxyl groups from H_2_O_2_, and also catalyzes the production of biliverdin from heme, of which the latter can form bilirubin, and biliverdin and bilirubin are powerful free radical scavengers in the body. It has been proved in mouse that γ-GCLase catalyzes the formation of GSH and is the most critical rate-limiting enzyme in GSH biosynthesis (254). Furthermore, OS results in ER stress, and Ufm1-specific ligase 1 (UFL1) alleviates cisplatin-induced apoptosis in ovarian GCs by attenuating ER stress, thereby relieving POI to some extent (254).The key to POI is the reduction in the quantity and quality of oocytes, which results in symptoms such as hot flashes, excessive sweating, vaginal dryness, and even causes serious consequences such as infertility.

## 4 Conclusion

The key to POI is the reduction in the quantity and quality of oocytes, which results in symptoms such as hot flashes, excessive sweating, vaginal dryness. Accordingly, it even causes serious consequences such as infertility. The role of apoptosis, abnormal meiosis, decreased mtDNA numbers, and ovarian microenvironment in POI is well known, but the mechanism of action is unclear. This review proposes that OS can mediate changes in genetic material, signaling pathways, transcription factors and ovarian microenvironment to accelerate oocyte and GCs senescence. Antioxidants are capable of counteracting the adverse effects of OS in the ovary and exhibit good therapeutic effects in the prevention and treatment of POI. We focus on exploring the mechanism of action of antioxidants, mesenchymal stem cells, and bioenzymes against OS in POI. Therefore, studying the relationship between OS and POI will not only help us understand the mechanism of action, but will also provide new insights into the protection of the female reproductive system and infertility.

## Data availability statement

The data of this article can be obtained with the permission of the corresponding author.

## Author contributions

Y-QS put forward the article idea and wrote the article. X-TZ wrote articles. S-NZ wrote articles. Y-FM translated and polished the manuscript. Y-HH revised the article. YJ provided technical support for the article. Y-HZ revised the article and provided financial support. All authors contributed to the article and approved the submitted version.

## Glossary

**Table:**

reactive oxygen species	ROS
oxidative stress	OS
premature ovarian insufficiency	POI
granulosa cells	GCs
mitochondrial DNA	mtDNA
mesenchymal stem cells	MSCs
European Society of Human Reproduction and Embryology	ESHRE
follicle stimulating hormone	FSH
endoplasmic reticulum	ER
perfluorononanoic acid	PFNA
hydrogen peroxide	H_2_O_2_
polyfluoroalkyl substances	PFASs
Perfluorohexanesulfonate	PFHxS
perfluorononanoate	PFNA
perfluorooctanoate	PFOA
perfluorooctanesulfonate	PFOS
Nivalenol	NIV
DNA damage response	DDR
double-strand breaks	DSBs
cyclophosphamide	CTX
deoxyribonucleoside triphosphates	dNTPs
Deltamethrin	DM
Bcl-2-associated X protein	Bax
B-cell lymphoma-2	Bcl-2
mitochondrial membrane potential	MMP
adenosine triphosphate	ATP
mitochondrial ribosomal protein L50	MRPL50
electron transport chain	ETC
mitochondrial transfer Ribonucleic acid	mt-tRNA
extracellular regulated kinase	ERK
microtubule organizing center	MTOC
metaphase II	MII
adenosine diphosphate	ADP
ADP-ribosyltransferase	ART
melatonin	MT
occult ovarian insufficiency	OOI
single-strand breaks	SSBs
telomeric repeat-containing RNA	TERRA
Phosphatidylinositol 3 kinase/protein kinase B	PI3K/Akt
mitogen-activated protein kinase	MAPK
Kelch-like ECH-associated protein 1	Keap1
nuclear factor erythroid 2-related factor 2	Nrf2
antioxidant response elements	ARE
Transforming growth factor	TGF
mammalian target of rapamycin	mTOR
forkhead box type O transcription factor-3a	FOXO_3a_
Bcl-xL BCL-2 associated death promoter	BAD
superoxide dismutase	SOD
glutathione peroxidase	GPX
glutathione	GSH
anti-Mullerian hormone	AMH
primordial follicles	PMFs
tuberous sclerosis complex 1	TSC1
tuberous sclerosis complex 2	TSC2
phosphatase and tensin homology deleted on chromosome 10	PTEN
Jun NH2 terminal kinase	JNK
big Mitogen-activated protein kinase 1	BMK1
mouse embryonic osteoblasts	MC3T3-E1
mitogen-activated protein kinase kinase 4	MKK4
mitogen-activated protein kinase kinase 7	MKK7
isocitrate dehydrogenase-1	IDH1
N-acetyl-L-cysteine/N-acetyl-l-cysteine	NAC
heme oxygenase-1	HO-1
nuclear factor-kappa B	NF-κB
quinone oxidoreductase	NQO1
red ginseng extract	RGE
D-galactose	D-gal
N6-methyladenosine	M^6^A
Bcl-2-associated athanogene 3	BAG-3
heat shock proteins 70	HSP70
Silent Information Regulator 3	SIRT3
murine double minute 2	MDM2
messenger RNA	mRNA
N1-methyladenosine	M^1^A
5-methylcytosine	M^5^C
methyltransferase-like 3	METTL3
obesity-associated protein	FTO
alkane hydroxylase	ALKB
ALKB homolog 5	ALKBH5
nicotinamide adenine dinucleotide	NAD
interleukin	IL
janus kinase	JAK
signal transducer and activator of transcription	STAT
tumor necrosis factor alpha	TNF-α
follicle-stimulating hormone receptor	FSHR
glucose-regulated protein 78	GRP78
Inducible nitric oxide synthase	iNOS
cyclooxygenase	COX
CC chemokine receptor 9	CCR9
C-X-C-motif chemokine receptor 3	CXCR3
stimulator of interferon genes	STING
vascular endothelial growth factor	VEGF
micro Ribose Nucleic Acid	miRNA
catalase	CAT
curcumin	CUR
Malondialdehyde	MDA
Ceramide	Cer
sphingosine-1-phosphate	S1P
coenzyme Q10	CoQ10
nicotinamide adenine dinucleotide phosphate oxidases	NOX
Resveratrol	RES
female germline stem cell	FGSC
estradiol	E2
follicle stimulating hormone	FSH
adipose mesenchymal stem cells	ADMSCs
placental mesenchymal stem cells	PMSCs
amniotic mesenchymal stem cells	AMSCs
umbilical cord mesenchymal stem cells	UCMSCs
amniotic fluid mesenchymal stem cells	AFMSCs
menstrual stem cells	MenSCs
nicotinamide adenine dinucleotide phosphate Quinone Dehydrogenase 1	NQO1
fetal mesenchymal stem cells	fMSCs
human mesenchymal stem cells derived from bone marrow	BMSC
insulin-like growth factor 1	IGF1
microRNA-17-5P	miR-17-5P
glutathione sulfhydryltransferase	GST
γ-Glutamate synthase	γ-GCL
Ubiquitin-like modifier 1 ligating enzyme 1	UFL1
randomised controlled trial	RCT
phosphate buffer solution	PBS
wild type	WT
human umbilical cord mesenchymal stem cells	hUMSCs
knockdown	KD
cisplatin	Cis
glucose-6-phosphate dehydrogenase	G6PDH
nicotinamide adenine dinucleotide phosphate	NADPH
cumulus oocyte complex	COC
luteinizing hormone	LH
mouse vasa homologue	Mvh
octamer-binding transcription factor 4	Oct4
tumor necrosis factor-α	TNF-α
malondialdehyde	MDA
senescence-associated beta	SA- β
P16 INhibitorofcyclin—dependentKinase4a	P16INK4a
Microtubule-associated protein light chain	LC3II
lactate dehydrogenase	LDH
glutathione reductase	GR
melatonin membrane receptor 1	MT1
Proliferation cell nuclear antigen	PCNA
AMP-activated protein kinase	AMPK
human umbilical cord mesenchymal stem cells	hUMSCs
human granulosa cells	hGCs
NADPH oxidases 4	NOX4
